# Carnosic Acid Mitigates Early Brain Injury After Subarachnoid Hemorrhage: Possible Involvement of the SIRT1/p66shc Signaling Pathway

**DOI:** 10.3389/fnins.2019.00026

**Published:** 2019-03-05

**Authors:** Lingfang Teng, Linfeng Fan, Yujiang Peng, Xijun He, Huihui Chen, Hongyu Duan, Fan Yang, Da Lin, Zheng Lin, Huiyong Li, Bo Shao

**Affiliations:** ^1^Department of Neurosurgery, The First People’s Hospital of Wenling, Wenling, China; ^2^Department of Pediatric Surgery, Capital Institute of Pediatrics, Beijing, China

**Keywords:** subarachnoid hemorrhage, carnosic acid, p66shc, early brain injury, apoptosis

## Abstract

Carnosic acid (CA) has been reported to exhibit a variety of bioactivities including antioxidation, neuroprotection, and anti-inflammation; however, the impact of CA on subarachnoid hemorrhage (SAH) has never been elucidated. The current study was undertaken to explore the role of CA in early brain injury (EBI) secondary to SAH and the underlying mechanisms. Adult male Sprague-Dawley rats were perforated to mimic a clinical aneurysm with SAH. CA or vehicle was administered intravenously immediately after the SAH occurred. Mortality, SAH grade, neurologic function scores, brain water content, Evans blue extravasation, and the levels of reactive oxygen species (ROS) levels in the ipsilateral cortex were determined 24 h after the SAH occurred. Western blot, immunofluorescence, Fluoro-Jade C (FJC) and TUNEL staining were also performed. Our results showed that CA decreased ROS levels, alleviated brain edema and blood-brain barrier permeability, reduced neuronal cell death, and promoted neurologic function improvement. To probe into the potential mechanisms. We showed that CA increased SIRT1, MnSOD, and Bcl-2 expression, as well as decreased p66shc, Bax, and cleaved caspase-3 expression. Interestingly, sirtinol, a selective inhibitor of SIRT1, abolished the anti-apoptotic effects of CA. Taken together, these data revealed that CA has a neuroprotective role in EBI secondary to SAH. The potential mechanism may involve suppression of neuronal apoptosis through the SIRT1/p66shc signaling pathway. CA may provide a promising therapeutic regimen for management of SAH.

## Introduction

Subarachnoid hemorrhage (SAH) is considered to be one of the most devastating cerebrovascular accidents, leading to a >50% combined morbidity and mortality rate (Chen et al., 2014b). Previous studies have attached importance to the vasospasm secondary to SAH, which is thought to mainly account for the delayed neurologic deficits that occur; however, clinical trials aimed at anti-vasospasm treatment have failed to promote the prognosis of SAH patients (Sehba et al., 2012; Sehba and Friedrich, 2015; Fan et al., 2017). The collective evidence indicates that early brain injury (EBI) contributes to the outcome of SAH. Although the specific mechanisms underlying EBI are controversial, neuronal apoptosis is deemed to have a fatal role in the process and might explain the short and long-term severe pathology of the disease (Biller et al., 1988; Cahill et al., 2006). Thus, extra effort is needed to develop an original and drug targeting apoptosis, which may provide a therapeutic regimen for SAH management.

Apoptosis is a complicated progress and multiple factors have been reported to trigger the process. Reactive oxygen species (ROS) are major causative factors inducing apoptosis (Redza-Dutordoir and Averill-Bates, 2016; Wu et al., 2017; Xia et al., 2018). SIRT1 is a well-characterized member of the highly conserved (NAD^+^)-dependent class III histone deacetylases and it could restrained ROS and apoptosis (Langley et al., 2002; Brunet et al., 2004). It has been demonstrated that overexpression of SIRT1 protects cardiomyocytes from oxidative injury (Cheng et al., 2003; McBurney et al., 2003; Xie et al., 2012). We previously reported that activated SIRT1 exhibited neuroprotection in SAH (Zhang X.S. et al., 2016); however, the underlying mechanism remains to be elucidated. P66shc, an isoform of the adapter protein ShcA, is a redox enzyme that can stimulate ROS generation and induce apoptosis (Giorgio et al., 2005; Galimov, 2010). Consistently, P66shc knockout mice have been reported to exhibit 30% longer lifespan and exert intensive resistance to oxidant stress (Berry et al., 2007; Kumar et al., 2014; Vikram et al., 2014). Recent studies suggested that p66shc may be regulated by SIRT1 (Chen et al., 2013; Shan et al., 2015). In SIRT1 transgenic diabetic mice, a decreased expression of p66shc has been observed (Zhou et al., 2011). Therefore, we hypothesize that SIRT1-mediated p66shc suppression may contributed to the prevention of SAH-induced brain injury.

Recently, more and more attentions have been paid to natural herbs in the treatment of SAH. Carnosic acid (CA), one of the dominant phenolic compounds in rosemary and sage leaves exhibits various pharmacologic properties, including antiapoptotic, antioxidant and chemopreventive activities (Shan et al., 2015). CA has been shown exhibit a protective effect against various tissue injuries by suppressing apoptosis (Xie et al., 2012; Chen et al., 2013; Shan et al., 2015). It has been reported that SIRT1 can be activated by plant polyphenols, such as resveratrol and quercetin (Chung et al., 2010). Likewise, SIRT1 may also be activated by CA. In this study, we sought to investigate the antiapoptotic effect of CA on protecting against SAH. And the regulation of SIRT1/p66shc pathway involved in CA-mediated protective activity in SAH was also explored.

## Materials and Methods

### Animals and Cell Lines

#### Animals

Adult male Sprague-Dawley rats (Slac Laboratory Animal Company Limited, Shanghai, China) weighting between 300 and 320 g (6–8 weeks of age) were used in the current study. The animals were housed under controlled temperature and humidity conditions with a 12 h light/dark cycles. All experiments were approved by the Institutional Ethics Committee of Zhejiang Province and consistent with the Guide for the Care and Use of Laboratory Animals of the National Institutes of Health and Animal Research.

#### Cell Culture

PC12 cells were obtained from American Type Culture Collection (ATCC, United States). Cells were maintained in DMEM supplemented with 10% FBS, 30 μg/ml penicillin, and 100 μg/ml Streptomycin at 37°C under a 5% CO_2_ atmosphere. For chemical treatment, cells were seed in 6-well plates and treated with 10 μM CA alone or together with 15 μM sirtinol for 24 h.

### SAH Model

The rat model were established as previously described (Li et al., 2016). Briefly, the rats were anesthetized with 40 mg/kg of pentobarbital sodium intraperitoneally. Second, the left carotid artery and its branches were dissected. A blunted 4-0 monofilament nylon suture was stabbed into the internal carotid artery from the external carotid artery and stopped until resistance appeared. Then the bifurcation of the anterior and middle cerebral arteries was punctured. Sham rats underwent a similar procedure without the vessel puncture. Finally, the suture was withdrawn after approximately 15 s. All rats were maintained at 37.5°C on a heating pad with rectal thermometer.

### Study Design

#### Experiment 1

To character the time course of p66shc after SAH, we detected the protein using a western blot assay in sham and SAH models for 1, 3, 6, 12, 24, 48, and 72 h (*n* = 6). Additionally, immunofluorescence co-staining was performed to localize p66shc in SAH rats (*n* = 6).

#### Experiment 2

One hundred twenty rates (163 rats were used and 43 rats died) were randomly allocated into four groups: sham (*n* = 30), SAH (*n* = 30/45), SAH + vehicle (*n* = 30/44), and SAH + CA (*n* = 30/44). The SAH group, the SAH + vehicle group and the SAH + CA groups were subjected to SAH. In addition, SAH + vehicle group and SAH + CA groups were treated with vehicle and CA, respectively. A similar procedure to that used in the SAH group was performed in the sham group but without perforation. All rats were evaluated 24 h after SAH was induced. SAH grade, neurologic score, brain water content, and Evans blue extravasation, and ROS assay, TUNEL staining, FJC staining, and Western blot analysis results were determined in each group.

#### Experiment 3

Seventy-two rats (107 rats were used and 35 rats died) were randomly assigned into 4 groups at random: SAH + vehicle group (*n* = 18/28), SAH + CA group (*n* = 18/26), SAH + sirtinol group (*n* = 18/27) and SAH + CA + sirtinol group (*n* = 18/26). Rats in the SAH + vehicle, the SAH + CA and the SAH + sirtinol group were exposed to SAH and treated with vehicle, CA, and sirtinol, respectively. The SAH + CA + sirtinol group was exposed in SAH and dealt with CA and sirtinol. The end point was 24 h after SAH. Brain water content, and Western blot analysis, FJC staining findings and TUNEL staining were determined in each group, respectively.

### Drug Administration

Carnosic acid was purchased from Tokyo Chemical Industry (Tokyo, Japan) and dissolved in dimethyl sulfoxide (DMSO). The dose and the time point of CA was chose according to a previous study (Miller et al., 2015). Vehicle (0.5% DMSO in a 10% Ethanol/90% PBS solution) or CA (3 mg/kg in a 10% Ethanol/90% PBS vehicle solution) were administrated intraperitoneally immediately after SAH. CA and its vehicle were administered 24 h prior to tissue collection. Sirtinol (Sigma-Aldrich, St. Louis, MO, United States) was administered via intracerebroventricular injection as previously described (Yan et al., 2016; Li et al., 2018). In brief, a small burr hole was drilled into the skull (1.5 mm posterior and 1.0 mm lateral relative to the bregma) after the rats were anesthetized. A 10 μl Hamilton syringe needle (Microliter 701; Hamilton Company, Reno, NV, United States) needle was inserted into the left lateral ventricle through the hole at a depth of (3.5 mm below the horizontal plane of the bregma). Sirtinol (a SIRT1 inhibitor) was dissolved in DSMO and further diluted in sterile saline to a final DSMO concentration of 0.5% [the dose of sirtinol was selected based on previous study (Zhang X.S. et al., 2016)]. Either sirtinol or vehicle was injected into the left lateral ventricle 2 h before SAH. The syringe was left *in situ* for at least 10 min before removal to prevent backfilling and then the hole was filled with bone wax.

### SAH Grades and Neurologic Scores

The severity of the SAH was evaluated using the SAH grading scale as previously described (Sugawara et al., 2008). In brief, the basal cisterns were allocated into 6 segments and each segment was scored from 0 to 3 based on the amount of bleeding as follows: grade 3, blood clots covered all arteries; grade 2, mediocre blood with visible arteries; grade 1, minimal subarachnoid blood; and grade 0, no SAH. We determined the total score by summing each segment score. We evaluated neurologic function 24 h after SAH according to the modified Garcia score (Garcia et al., 1995). Evaluation of autonomic exercise, exercise coordination, physical activity, and somatic sensation was included. The score ranged from 3 to 18. Six tests including response to vibrissa touch, limb symmetry, body proprioception, climbing, spontaneous activity, and forelimb outstretching were scored and total scores were measured. An independent observer performed all evaluation.

### Brain Water Content

The right and left hemispheres of the brains were removed after the rats were euthanized. Each part of the brain was weighed immediately upon removal (wet weight), then put in an oven at 105°C to dry. The brain parts were re-weighed after 72 h (dry weight). The brain water content was computed as follows: (wet weight-dry weight)/wet weight × 100%.

### Evans Blue Extravasation

Evans blue could combine with plasma albumin and permeate into the brain tissues by the disruptive BBB. So the permeability of the blood-brain barrier (BBB) was assessed according to Evans blue extravasation. Evans blue extravasation was performed as previously reported (Chen et al., 2014a). The left femoral vein was injected with Evans Blue dye (2%, 5 mL/kg) under general anesthesia 24 h after surgery. After circulating for 60 min, the rats were euthanized, then perfused with 0.01 mol/L phosphate-buffered saline (PBS). The brain was immediately removed and separated into the same regions. We weighed and homogenized the samples in 3 ml of 50% trichloroacetic acid, then centrifuged the samples at 15000 ×*g* for 30 min. The supernatant (1 ml) was separated and mixed with an equal volume of the mixture (1:3 trichloroacetic acid and ethanol). Then the samples were centrifuged again for 30 min after a 12 h incubation at 4°C. Then, we assessed the supernatant through spectrophotometry (620 nm for excitation and 680 nm for emission).

### ROS Assay

The left basal cortical specimen in the face of the blood clot was collected at 24 h after SAH. We used a ROS assay kit (Nanjing Jiancheng Bio-engineering Institute, Nanjing, China) to detect the ROS levels of the rats’ brains referring to the manufacturer protocol. Briefly, rats were perfused with 0.01 mol/L PBS after euthanasia. We subsequently obtained fresh tissues from the brains. Then, the samples were weighed and homogenized in PBS (1 g: 20 ml). The mixtures were centrifuged at 1000 ×*g* for 10 min at 4°C and measured the protein content of the supernatant with a DC protein assay kit (Bio-Rad, Hercules, CA, United States). According to the protocol, the DCFH-DA (10 μl, 1 mol/L) and supernatant (190 μl) were mixed into 96-well plates and the same volume of PBS was added to the supernatant as a control. The samples were detected by spectrofluorophotometry after incubation for 30 min 3°C, 480 nm excitation wavelength and 520 nm emission wavelength. The ROS levels are presented as fluorescence intensity/gram protein (Li et al., 2016).

### Immunofluorescence, TUNEL, and Fluoro-Jade C (FJC) Staining

Rats were perfused transcardially with PBS (0.1 mol/L) followed by 4% paraformaldehyde and euthanized 24 h after SAH. The brains of each group (*n* = 6) were gained and dipped in the 4% PFA for 24 h, and then dehydrated with sucrose solution (30%). The brains were frozen in tissue-freezing media to cut into coronary sections (7 μm). Conforming to the immunofluorescence protocol, we washed the coronal sections with 0.01 M PBS 3 times and then blocked with 10% normal goat serum (with 0.1% Triton X-100 in 0.01 M PBS) sealing solution. Subsequently the sections were incubated overnight with relevant primary antibodies including anti-p66shc (ab54518, Abcam), anti-NeuN (ab177487, Abcam). After washed with PBS several times the sections were hatched with related secondary antibodies including fluorescein isothiocyanate-labeled goat anti-mouse antibody (Jackson ImmunoResearch) and rhodamine-conjugated goat anti-rabbit antibody (Jackson ImmunoResearch). The sections were stained with DAPI after washing again, then mounted with glycerol. We observed the sections using a fluorescent microscope (Olympus, Tokyo, Japan) and merged the photomicrographs by Image-Pro Plus 6.0 (Olympus, Melville, NY, United States). Five random files per coverslip were imaged. We used FJC staining to identify degenerating neurons according to the manufacturer’s instructions for the FJC staining kit (Biosensis, NY, United States). In addition, terminal deoxynucleotidyl transferase-dUTP nick end labeling (TUNEL) staining was also applied to determine apoptotic cell according to the manufacturer’s protocol (Roche Inc., Basel, Switzerland). An independent investigator counted FJC and TUNEL positive neurons in the left piriform cortex.

### SIRT1 Activity

SIRT1 activity in brain was determined with a SIRT1 Fluorometric Kit (Biomol International) according to the manufacturer’s instructions and as described previously (Escande et al., 2010). This assay uses a small lysine-acetylated peptide, corresponding to K382 of human p53, as a substrate. SIRT1 could deacetylate the lysine residue, and this process is dependent on the addition of exogenous NAD^+^. Briefly, samples were homogenized in NETN buffer and then incubated for 10 min at 37°C. Next, 10 mM DTT was added to the medium and the mixtures were incubated for 10 min at 37°C again. The mixtures (20–30 μg protein/well) were then incubated in SIRT1 assay buffer to determine the SIRT1-independent or SIRT1-dependent activity. After 1 h incubation, the reaction was terminated by adding the solution containing Fluor de Lys Developer (Enzo Life Sciences) and 2 mM nicotinamide. And then the mixtures were incubated for 60 min at 37°C. The samples were detected with an excitation wavelength of 360 nm and an emission wavelength of 460 nm (Spectramax Gemini XPS; Molecular Devices). The SIRT1-dependent activity was assessed after subtracting fluorescence values obtained in the absence of NAD^+^.

### Western Blot

We performed Western blot as previously described (Zhang X.S. et al., 2016). Briefly, samples were obtained from cells or the left basal cortical, then lyzed on ice or homogenized and centrifuged (1000 ×*g*) for 10 min at 4°C. A DC protein assay kit (Bio-Rad, Hercules, CA, United States) was used to detect the protein content. Equal amounts (60 μg) of proteins were added into polyacrylamide-SDS gels. The proteins were transferred to nitrocellulose membranes after separated by electrophorese. Membranes were incubated overnight at 4°C with the following primary antibodies followed by sealing with non-fat dry milk buffer: anti-p66shc (ab54518, Abcam), anti-SIRT1 (ab110304, Abcam), anti-ß-actin (ab8226, Abcam), anti-MnSOD (ab13533, Abcam), anti-Bax (ab32503, Abcam), anti-Bcl-2 (ab32124, Abcam), and anti-caspase-3 (ab13585, Abcam). Then, the membranes were incubated with the corresponding secondary antibodies at room temperature for 1h. The protein densities were detected via X-ray film and quantified with ImageJ software (NIH).

### Statistical Analysis

Data were expressed as mean ± SD or median with interquartile range. The analyses were carried out using GraphPad Prism 7 (GraphPad Software Inc., San Diego, CA, United States) and SPSS (version 24.0; SPSS, Inc., Chicago, IL, United States). We used one-way analysis of variance (ANOVA) followed by Tukey’s multiple comparison test to analyze differences among the groups when the data met the normal distribution and homogeneity of variance. For the non-normal distribution and unequal variance parameters, differences among the groups were analyzed using Kruskal–Wallis test. The Garcia score and SAH grading score were analyzed using the Mann–Whitney *U*-test. Statistical significance was defined as *P* < 0.05.

## Results

### Expression of p66shc After SAH

**FIGURE 1:**
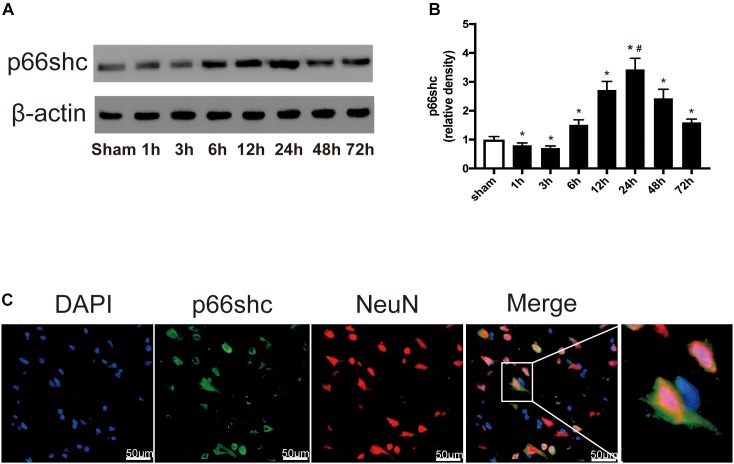
**(A)** Representative Western blot bands of p66shc at different time course in ipsilateral basal cortex after subarachnoid hemorrhage (SAH) induction. **(B)** Quantitative analysis of p66shc expression. *n* = 6 for each group. The bars represent the mean ± SD. ^∗^*P* < 0.05 versus sham, ^#^*P* < 0.05 versus every other group. The densities of the protein bands were analyzed in relation to β-actin and normalized to the sham group. **(C)** Representative microphotographs of immunofluorescence staining showing localization of p66shc (green) with NeuN (red) in ipsilateral basal cortex of SAH 24 h group (scale bar = 50 μm). The rightmost image was magnified by digital zoom.

The expression of p66shc in the sham group and in rats euthanized at 1, 3, 6, 12, 24, 48, and 72 h after SAH was detected by Western blot (Figure 1A). The result showed that the expression of p66shc was reduced at 1 and 3 h after SAH, but began to increase at 6 h and peaked at 24 h (*P* < 0.05, Figure 1A,B). Immunofluorescence co-staining of p66shc with NeuN (the marker of neurons) confirmed that p66shc was localized in neurons (Figure 1C).

### Mortality, SAH Grade, and Neurologic Dysfunction

**FIGURE 2:**
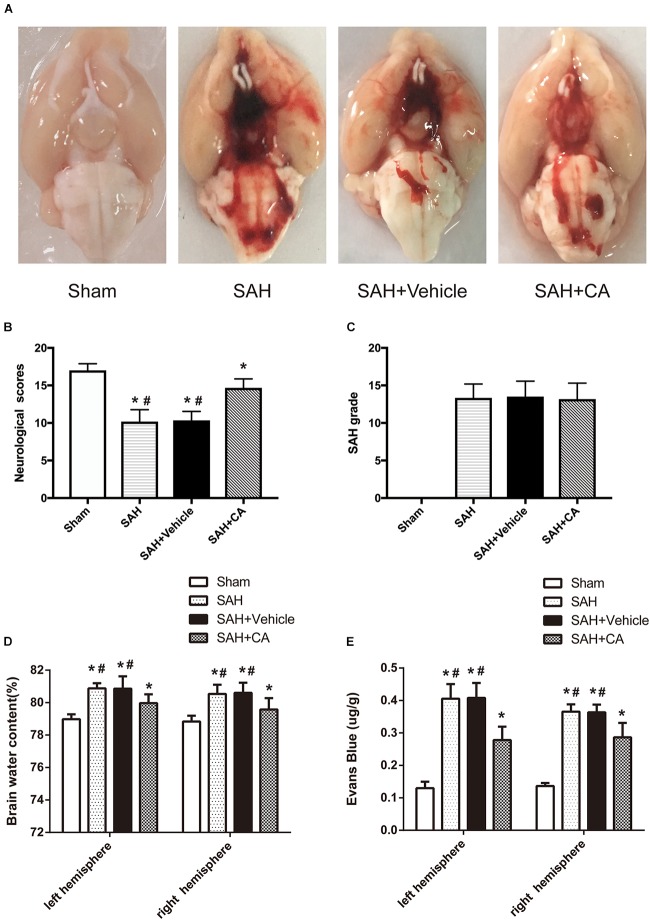
Typical representation of brains from each group and SAH grade, neurological scores, brain water content, and Evans blue dye extravasation at 24 h after SAH. **(A)** Representative brains from the sham, SAH, SAH + vehicle, SAH + CA group. **(B)** Quantitative analyses of neurological scores. The bars represent the mean ± SD. *n* = 30. **(C)** Quantitative analyses of SAH severity. The bars represent the mean ± SD. *n* = 30. **(D)** Quantitative analyses of brain water content. The bars represent the mean ± SD. *n* = 6. **(E)** Quantitative analyses of Evans blue dye extraversion. The bars represent the mean ± SD. *n* = 6. ^∗#^*P* < 0.05 vs. Sham, ^∗^*P* < 0.05 vs. SAH.

Representative brains from the sham, SAH, SAH + vehicle, and SAH + CA groups are presented in Figure 2A. More blood clots were found in the brain of the SAH group compared with the Sham group, and treatment with CA significantly reduced the blood clots. The neurological scores showed that significant neurological impairments occurred in SAH and SAH + vehicle groups when compared with the sham group. Administration of CA significantly improved neurological impairments 24 h after SAH (*P* < 0.05, Figure 2B). There was no significant difference among the mortality rates of the different groups. There were no significant differences in SAH grade between the SAH + vehicle and SAH + CA groups (*P* > 0.05, Figure 2C).

### CA Ameliorated Brain Edema and Disruption of BBB

We observed great changes of the brain water content at 24 h after SAH. The brain water content of the left and right cerebral hemisphere increased in SAH and SAH + vehicle group in comparison with the sham group (*P* < 0.05, Figure 2D). After CA treatment, the brain water content in the left and right hemisphere was notably reduced compared with SAH + vehicle group (*P* < 0.05, Figure 2D). A remarkable extravasation of Evans blue dye into the left and right hemispheres was found in SAH and SAH + vehicle group in comparison with the sham group (*P* < 0.05, Figure 2E). CA significantly decreased Evans blue dye extravasation in the left and right hemispheres (*P* < 0.05, Figure 2E).

### Administration of CA Activated the SIRT1/p66shc Pathway and Inhibited Apoptosis

**FIGURE 3:**
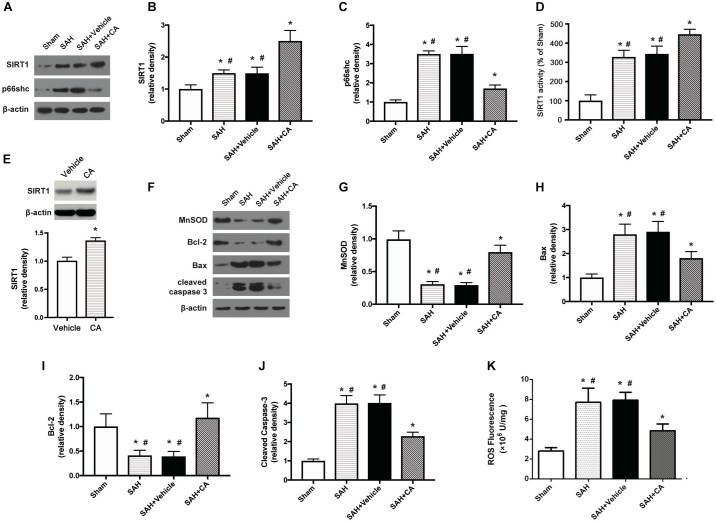
Carnosic acid attenuated apoptosis 24 h after subarachnoid hemorrhage (SAH) via activating SIRT1/p66shc signaling pathway. **(A)** Representative Western blot bands of SIRT1, p66shc. **(B,C)** Quantitative analyses of SIRT1 **(B)**, p66shc **(C)**. **(D)** Activity of SIRT1 in the brain. **(E)** The effect of CA on SIRT1 expression. **(F)** Representative Western blot bands of MnSOD, Bax, Bcl-2, and cleaved caspase-3. **(G–J)** Quantitative analyses of, MnSOD **(G)**, Bax **(H)**, Bcl-2 **(I)**, cleaved caspase-3 **(J)**. The densities of the protein bands were analyzed in relation to β-actin and normalized to the sham group. **(K)** Quantitation of ROS. The histograms represent the median with interquartile range, *n* = 6. The statistical differences between two groups were analyzed by Kruskal–Wallis test. ^∗#^*P* < 0.05 vs. Sham, ^∗^*P* < 0.05 vs. SAH.

Carnosic acid was administered intraperitoneally after the SAH was induced. Western blot analysis showed that administration of CA significantly upregulated the expression of SIRT1 and downregulated the expression of p66shc compared to the SAH and SAH + vehicle groups (*P* < 0.05, Figure 3A–C). Consistent with the result from western blot, the activity of SIRT1 was significantly increased after SAH, and this increase was further extended by administration of CA (*P* < 0.05, Figure 3D). To determine the direct effect of CA on SIRT1 expression, PC12 cells were treated with CA. Western blot analysis showed that treatment with CA significantly increased SIRT1 expression (Figure 3E). The expression of MnSOD, a mitochondria-resident enzyme that governs ROS, was significantly decreased after SAH induction and upregulated after CA administration (*P* < 0.05, Figure 3F,G). Apoptosis associated proteins, such as Bax, Bcl-2, and cleaved caspase-3 were dramatically altered 24 h after SAH compared with the sham group, and the changes were reversed by CA administration (*P* < 0.05, Figure 3H–J). In addition, levels of ROS were remarkably increased after SAH, whereas they were markedly reduced in the SAH + CA group (*P* < 0.05, Figure 3K).

**FIGURE 4:**
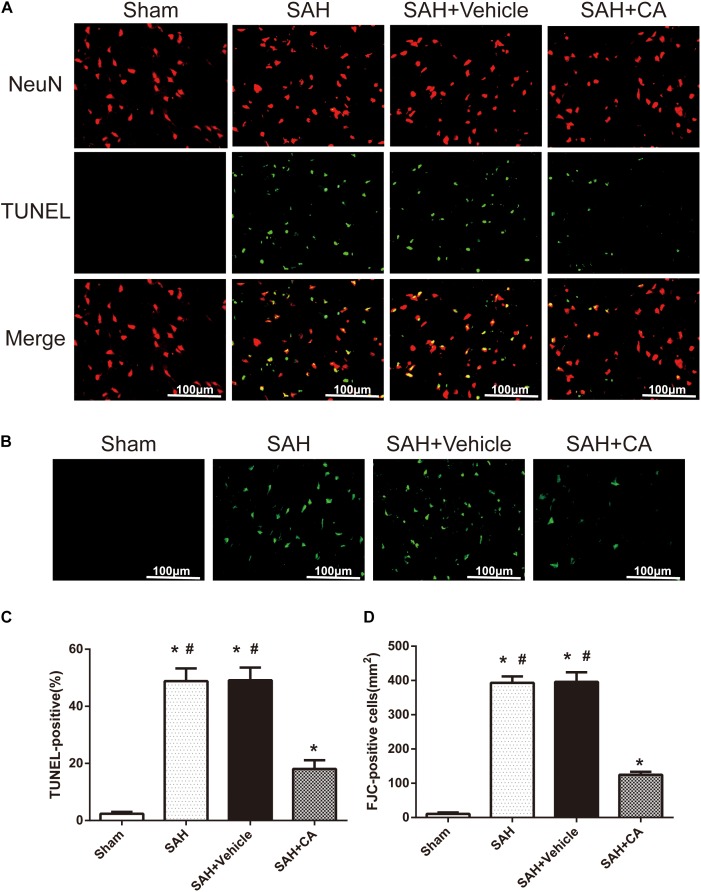
Carnosic acid reduced degenerating neurons and neuronal cells death in the ipsilateral cortex at 24 h after SAH. **(A)** Representative co-labeling TUNEL/NeuN photomicrographs of the ipsilateral cortex in the different groups (scale bar = 100 μm). **(B)** Representative FJC photomicrographs in the different groups (scale bar = 100 μm). **(C)** Quantification of TUNEL-positive neurons, expressed as percentage of total (NeuN^+^) cells. **(D)** Quantification of FJC-positive degenerating neurons. The bars represent the mean ± SD. *n* = 6. ^∗#^*P* < 0.05 vs. Sham, ^∗^*P* < 0.05 vs. SAH.

Few TUNEL-positive neurons were detected in the sham group after SAH. The numbers of TUNEL-positive neurons in the cortex in the SAH and SAH + vehicle group were remarkably higher compared with the sham group. In comparison with the SAH and SAH + vehicle groups, CA treatment decreased the apoptosis index (*P* < 0.05, Figure 4A,C). FJC-positive cells representing degenerating neurons dramatically increased after SAH and CA treatment reversed this change (*P* < 0.05, Figure 4B,D). These results suggest that CA treatment inhibits neuron apoptosis and SIRT1/p66shc cascade might account for this inhibition.

### Inhibition of SIRT1 Abolished the Neuroprotective Effect of CA

**FIGURE 5:**
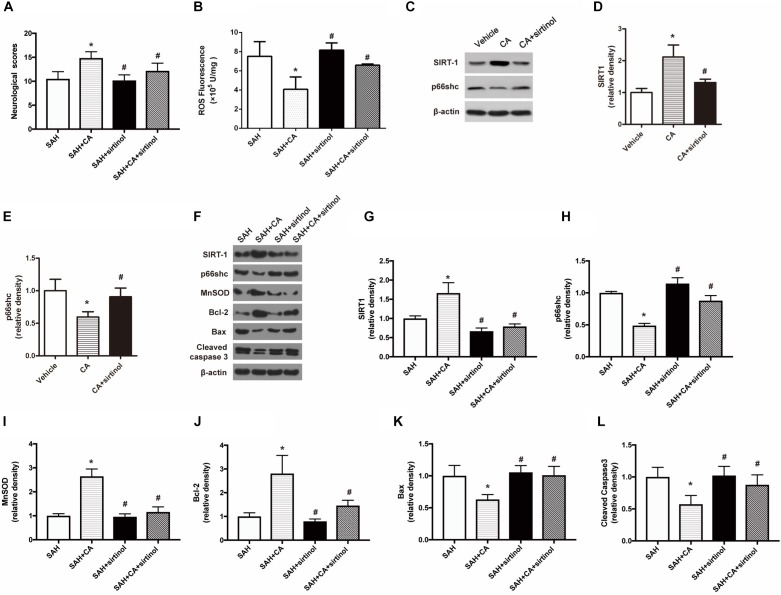
SIRT1 inhibitor sirtinol abolished the beneficial effect of carnosic acid on apoptosis. **(A)** Quantitative analyses of neurological scores. The bars represent the mean ± SD. *n* = 18. **(B)** Quantitative analyses of ROS. The histograms represent the median with interquartile range, *n* = 6. **(C–E)** Western blot assay and quantitative analyses of SIRT1 **(D)**, p66shc **(E)** in PC12 cells after treatment with CA alone or together with sirtinol. ^∗^*P* < 0.05 vs. Vehicle, ^#^*P* < 0.05 vs. CA. **(F–L)** Western blot assay **(F)** and quantitative analyses of SIRT1 **(G)**, p66shc **(H)**, MnSOD **(I)**, Bcl-2 **(J)**, Bax **(K)**, cleaved caspase-3 **(L)** expressions in ipsilateral cortex. The densities of the protein bands were analyzed in relation to β-actin and normalized to the sham group. The bars represent the mean ± SD. *n* = 6. ^∗^*P* < 0.05 vs. SAH, ^#^*P* < 0.05 vs. SAH + CA.

To test whether SIRT1 upregulation plays a causal role in CA-mediated neuroprotection, sirtinol, a selective inhibitor of SIRT1, was applied. The results showed that inhibition of SIRT1 by sirtinol significantly abolished the neurological improvements induced by CA, as indicated by the neurological scores (*P* < 0.05, Figure 5A). Additionally, the levels of ROS rebounded after sirtinol administration (*P* < 0.05, Figure 5B). In PC12 cells, treatment with sirtinol abolished the inhibitor effect of CA on p66shc inhibition (*P* < 0.05, Figure 5C–E). Moreover, Western blot showed that upregulation of SIRT1 and downregulation of p66shc caused by CA were also reversed by sirtinol (*P* < 0.05, Figure 5F–H).

### Inhibition of SIRT1 Reversed the Anti-apoptotic Effect of CA After SAH

As shown in the Western blot analysis 24 h after SAH, administration of CA significantly increased MnSOD expression and the tendency was notably reversed in the SAH + CA + sirtinol group (*P* < 0.05, Figure 5I). Upregulation of Bcl-2 in the SAH + CA group was also dramatically suppressed by sirtinol (*P* < 0.05, Figure 5J). Sirtinol also reversed the CA-induced decrease of Bax and cleaved caspase-3 expression (*P* < 0.05, Figure 5K,L).

**FIGURE 6:**
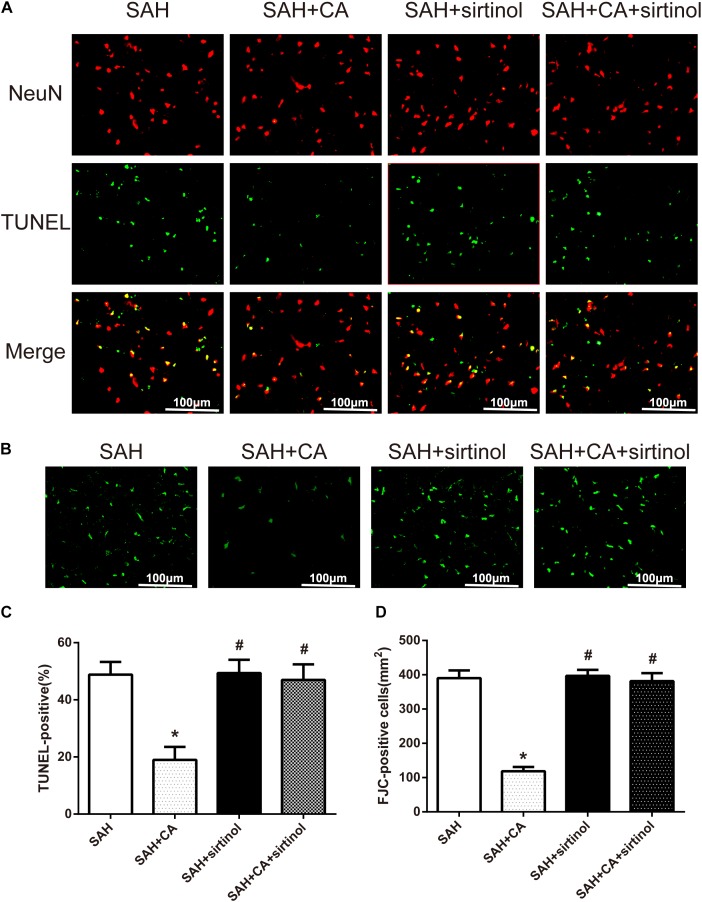
SIRT1 inhibitor sirtinol abolished Carnosic acid-reduced degenerating neurons and neuronal cells death in the ipsilateral cortex at 24 h after SAH. **(A)** Representative co-labeling TUNEL/NeuN photomicrographs of the ipsilateral cortex in the different groups (scale bar = 100 μm). **(B)** Representative FJC photomicrographs in the different groups (scale bar = 100 μm). **(C)** Quantification of TUNEL-positive neurons, expressed as percentage of total (NeuN^+^) cells. **(D)** Quantification of FJC-positive degenerating neurons. The bars represent the mean ± SD. *n* = 6. ^∗^*P* < 0.05 vs. SAH, ^#^*P* < 0.05 vs. SAH + CA.

Further, TUNEL and FJC staining were used to measure apoptotic cells and degenerating neurons in the ipsilateral cortex. Results showed that treatment of CA significantly decreased the apoptosis index and FJC-positive cells. However, these alterations were markedly reversed by sirtinol (*P* < 0.05, Figure 6A–D).

## Discussion

Mounting evidence implies that EBI within the first 72 h of SAH plays a central role in SAH. The treatment of EBI could be a potential therapeutic strategy for the management of patients surviving a SAH. However, the underlying molecular mechanisms has not been elucidated (Ostrowski et al., 2006). The possible mechanisms involving EBI after SAH involve disruption of the BBB, brain edema, oxidative stress, and neural apoptosis (Sehba et al., 2012). CA, one of the major phenolic compounds extracted from *Rosmarinus officinalis*, is a well-established anti-adipogenic and antioxidant agent (Jordan et al., 2012; Park and Mun, 2013). Recently, CA has been reported to have favorable efficacy in managing some diseases. For example, CA protects myocardial cells, renal cells, SH-SY5Y cells, and hepatocytes against injury by inhibiting apoptosis (Sahu et al., 2011, 2014; Chen J.H. et al., 2012). The mechanisms by which CA regulates neural apoptosis in SAH is unknown. In a recent study, we successfully established the vascular perforation model to mimic clinical aneurysm subarachnoid hemorrhage (aSAH), which is the most common cause of SAH. We observed that CA treatment alleviated neuronal cell death and improved neurological outcome, diminished levels of ROS, and dramatically alleviated BBB disruption and brain edema dramatically. We therefore suggest that the SIRT1/p66shc pathway might be involved in the protective mechanism underlying CA treatment of SAH.

Emerging evidence indicates that under pathophysiologic circumstances, acute and chronic overproduction of ROS are vitally important in the development of cardio-cerebral vascular diseases. ROS, which are highly reactive free radicals including a variety of small molecule radicals such as superoxide anion (O_2_), hydroxyl radical (OH), and hydrogen peroxide (H_2_O_2_), which are important components of cellular signaling pathways physiologically (Venditti et al., 2013; Fan et al., 2017). ROS are also the most important cause of oxidative stress. The increased levels of ROS could incur brain injury by various means, including apoptosis, BBB disruption, and inflammation (Cahill et al., 2006; Olmez and Ozyurt, 2012; Sehba and Friedrich, 2015; Zhang L. et al., 2016). Thus, we measured the levels of ROS in recent study. The cortical ROS levels surged 24 h after SAH and were inhibited by CA. This result further verified the antioxidative activity of CA. To further explore the potential mechanism underlying antioxidative stress by CA, we focused on SIRT1, an NAD-dependent deacetylase. Recently, it has been demonstrated that activation of SIRT1 plays a critical protective role in mediating tissue injury in multiple experimental models and human diseases including cardiac, brain and kidney injuries caused by ischemia/reperfusion (Hsu et al., 2010; Wang et al., 2011; Lempiainen et al., 2012). Interestingly, SIRT1 is widely expressed in the central nervous system and SIRT1 is activated by plant polyphenols. Consistent with our hypothesis, the current study determined that CA treatment facilitated the activation of SIRT1 after SAH and the positive regulatory effect of CA on SAH was restrained by sirtinol, a SIRT1 inhibitor. These results indicate that CA might be a potential activator of SIRT1. Nevertheless, it is notable that the expression of SIRT1 increased slightly at 24 h after SAH. We speculate that self-protection against injury stress might be responsible for this result, which is supported by the results of a previous study (Li et al., 2018).

It has been shown that SIRT1 regulates energy metabolism, oxidative stress, and apoptosis by targeting different genes in the liver (Purushotham et al., 2009). P66shc has been reported to be targeted by SIRT1 through deacetylation of histone H3 lysine (Zhou et al., 2011). Moreover, it has been suggested that CA-promoted activation of the SIRT1/p66shc pathway protects rats against apoptosis in an ischemia/reperfusion (I/R) injury model (Yan et al., 2014). P66shc, an intracellular mediator converts oxidative signals into ROS and induces apoptosis during multiple pathologic conditions, such as diabetes and glomerulopathies (Menini et al., 2007; Zaccagnini et al., 2007). A P66shc defective mutant is unable to maintain normal oxidative stress in p66shc^-/-^ cells and p66shc knockout strengthen cellular resistance to apoptosis induced by H_2_O_2_ and ultraviolet light (Menini et al., 2007). Furthermore, p66shc has been suggested to downregulate the expression of antioxidant enzymes and regulatory factors, such as glutathione peroxidase-1, MnSOD (the primary superoxide scavenger) and REF-1 (Miyazawa and Tsuji, 2014). Interestingly, it has been shown that the expression of p66shc is decreased and oxidative stress is reduced markedly in the SIRT1 transgenic diabetic mice (Chen H. et al., 2012). In agreement with these observations, we found that CA treatment reversed the upregulation of p66shc caused by SAH and sirtinol induced an increase in p66shc. Interestingly, CA increased the levels of MnSOD after SAH and sirtinol abolished these changes. Together, these results imply that SIRT1 suppressed p66shc expression and oxidative stress during SAH and that activation of the SIRT1/p66shc pathway might be involved in the neuroprotective effect of CA in SAH.

Current views hold that the formation of a multi-subunit protein complex, which eventually forms a pore in the mitochondrial membrane is promoted by various signals in the course of mitochondrial-dependent apoptosis. The permeabilization of mitochondrial membranes results in proapoptotic proteins activation. It has been demonstrated that p66shc is necessary for permeabilization of mitochondrial membranes (Galimov, 2010). The protective effects of SIRT1 against cardiac I/R can be explained by SIRT1 overexpression inducing up-regulation of MnSOD and Bcl-2 and down-regulation of Bax (Hsu et al., 2010). Similarly, suppression of p66shc protected against intestinal I/R induced by lung injury via the regulation of MnSOD, Bcl-2 and caspase-3 (Wang et al., 2012). In our study, FJC and TUNEL staining revealed that the neuronal degeneration and apoptosis secondary to SAH were suppressed by CA. Additionally, we also determined that the expression of Bax and cleaved caspase-3 were increased markedly after SAH, while CA treatment reserved the changes. The expression of Bcl-2 were presented on the contrary, namely, CA administration increased the expression of Bcl-2. Moreover, Sirtinol diminished the anti-apoptotic effect of CA on EBI. Based on these observations, we deduced that the activation of p66shc is strongly linked to apoptosis secondary to SAH and it is likely that CA protects neuron against apoptosis via suppression of the SIRT1/p66shc signaling pathway.

Several restrictions exist in the present study. First, we discussed the neuroprotection of CA in the early stage of SAH, the long-term effect in SAH needs to be further investigated. Second, several studies have revealed the effect of CA regulating inflammation. In our study, we focused on the anti-apoptosis effect of CA on SAH, however, we cannot completely absolutely exclude the possibility that neuroinflammation is involved in the neuroprotection of CA in EBI after SAH. Therefore, further studies are needed to explore the role of CA in neuroinflammation after SAH. In addition, ROS-induced oxidative stress after SAH warrants further experiments.

In conclusion, this study extended our understanding of the neuroprotective effects of CA on EBI after SAH. We demonstrated that CA-induced SIRT1 upregulation plays a protective role in EBI secondary to SAH. The potential mechanisms may involve the suppression of neuronal apoptosis through the SIRT1/p66shc pathway. Indeed, CA might be a novel and potential therapeutic strategy for SAH management.

## Author Contributions

LT and LF contributed to the study design, data analysis, and paper writing. YP, XH, HC, HD, FY, DL, ZL, and HL contributed to the SAH model performance and data collection. BS contributed to the instruction of the total research.

## Conflict of Interest Statement

The authors declare that the research was conducted in the absence of any commercial or financial relationships that could be construed as a potential conflict of interest.

## References

[B1] Berry A. Capone F. Giorgio M. Pelicci P. G. De Kloet E. R. Alleva E. (2007). Deletion of the life span determinant p66Shc prevents age-dependent increases in emotionality and pain sensitivity in mice. *Exp. Gerontol.* 42 37–45. 10.1016/j.exger.2006.05.018 16809014

[B2] Biller J. Godersky J. C. Adams H. P. Jr. (1988). Management of aneurysmal subarachnoid hemorrhage. *Stroke* 19 1300–1305. 10.1161/01.STR.19.10.1300 3176090

[B3] Brunet A. Sweeney L. B. Sturgill J. F. Chua K. F. Greer P. L. Lin Y. (2004). Stress-dependent regulation of FOXO transcription factors by the SIRT1 deacetylase. *Science* 303 2011–2015. 10.1126/science.1094637 14976264

[B4] Cahill J. Calvert J. W. Zhang J. H. (2006). Mechanisms of early brain injury after subarachnoid hemorrhage. *J. Cereb. Blood Flow Metab.* 26 1341–1353. 10.1038/sj.jcbfm.9600283 16482081

[B5] Chen H. Wan Y. Zhou S. Lu Y. Zhang Z. Zhang R. (2012). Endothelium-specific SIRT1 overexpression inhibits hyperglycemia-induced upregulation of vascular cell senescence. *Sci. China Life Sci.* 55 467–473. 10.1007/s11427-012-4329-4 22744176

[B6] Chen J. H. Ou H. P. Lin C. Y. Lin F. J. Wu C. R. Chang S. W. (2012). Carnosic acid prevents 6-hydroxydopamine-induced cell death in SH-SY5Y cells via mediation of glutathione synthesis. *Chem. Res. Toxicol.* 25 1893–1901. 10.1021/tx300171u 22894569

[B7] Chen H. Z. Wan Y. Z. Liu D. P. (2013). Cross-talk between SIRT1 and p66Shc in vascular diseases. *Trends Cardiovasc. Med.* 23 237–241. 10.1016/j.tcm.2013.01.001 23499302

[B8] Chen J. Chen G. Li J. Qian C. Mo H. Gu C. (2014a). Melatonin attenuates inflammatory response-induced brain edema in early brain injury following a subarachnoid hemorrhage: a possible role for the regulation of pro-inflammatory cytokines. *J. Pineal Res.* 57 340–347. 10.1111/jpi.12173 25187344

[B9] Chen J. Wang L. Wu C. Hu Q. Gu C. Yan F. (2014b). Melatonin-enhanced autophagy protects against neural apoptosis via a mitochondrial pathway in early brain injury following a subarachnoid hemorrhage. *J. Pineal Res.* 56 12–19. 10.1111/jpi.12086 24033352

[B10] Cheng H. L. Mostoslavsky R. Saito S. Manis J. P. Gu Y. Patel P. (2003). Developmental defects and p53 hyperacetylation in Sir2 homolog (SIRT1)-deficient mice. *Proc. Natl. Acad. Sci. U.S.A.* 100 10794–10799. 10.1073/pnas.1934713100 12960381 PMC196882

[B11] Chung S. Yao H. Caito S. Hwang J. W. Arunachalam G. Rahman I. (2010). Regulation of SIRT1 in cellular functions: role of polyphenols. *Arch. Biochem. Biophys.* 501 79–90. 10.1016/j.abb.2010.05.003 20450879 PMC2930135

[B12] Escande C. Chini C. C. Nin V. Dykhouse K. M. Novak C. M. Levine J. (2010). Deleted in breast cancer-1 regulates SIRT1 activity and contributes to high-fat diet-induced liver steatosis in mice. *J. Clin. Invest.* 120 545–558. 10.1172/JCI39319 20071779 PMC2810074

[B13] Fan L. F. He P. Y. Peng Y. C. Du Q. H. Ma Y. J. Jin J. X. (2017). Mdivi-1 ameliorates early brain injury after subarachnoid hemorrhage via the suppression of inflammation-related blood-brain barrier disruption and endoplasmic reticulum stress-based apoptosis. *Free Radic. Biol. Med.* 112 336–349. 10.1016/j.freeradbiomed.2017.08.003 28790012

[B14] Galimov E. R. (2010). The Role of p66shc in oxidative stress and apoptosis. *Acta Nat.* 2 44–51. 10.1016/j.freeradbiomed.2017.08.003 22649663 PMC3347587

[B15] Garcia J. H. Wagner S. Liu K. F. Hu X. J. (1995). Neurological deficit and extent of neuronal necrosis attributable to middle cerebral artery occlusion in rats. Statistical validation. *Stroke* 26 627–634, discussion 635. 10.1161/01.STR.26.4.627 7709410

[B16] Giorgio M. Migliaccio E. Orsini F. Paolucci D. Moroni M. Contursi C. (2005). Electron transfer between cytochrome c and p66Shc generates reactive oxygen species that trigger mitochondrial apoptosis. *Cell* 122 221–233. 10.1016/j.cell.2005.05.011 16051147

[B17] Hsu C. P. Zhai P. Yamamoto T. Maejima Y. Matsushima S. Hariharan N. (2010). Silent information regulator 1 protects the heart from ischemia/reperfusion. *Circulation* 122 2170–2182. 10.1161/CIRCULATIONAHA.110.958033 21060073 PMC3003297

[B18] Jordan M. J. Lax V. Rota M. C. Loran S. Sotomayor J. A. (2012). Relevance of carnosic acid, carnosol, and rosmarinic acid concentrations in the in vitro antioxidant and antimicrobial activities of *Rosmarinus officinalis* (L.) methanolic extracts. *J. Agric. Food Chem.* 60 9603–9608. 10.1021/jf302881t 22957812

[B19] Kumar S. Vikram A. Kim Y. R. S Jacobs J. Irani K. (2014). P66Shc mediates increased platelet activation and aggregation in hypercholesterolemia. *Biochem. Biophys. Res. Commun.* 449 496–501. 10.1016/j.bbrc.2014.05.029 24845561

[B20] Langley E. Pearson M. Faretta M. Bauer U. M. Frye R. A. Minucci S. (2002). Human SIR2 deacetylates p53 and antagonizes PML/p53-induced cellular senescence. *EMBO J.* 21 2383–2396. 10.1093/emboj/21.10.2383 12006491 PMC126010

[B21] Lempiainen J. Finckenberg P. Levijoki J. Mervaala E. (2012). AMPK activator AICAR ameliorates ischaemia reperfusion injury in the rat kidney. *Br. J. Pharmacol.* 166 1905–1915. 10.1111/j.1476-5381.2012.01895.x 22324445 PMC3402813

[B22] Li J. Chen J. Mo H. Chen J. Qian C. Yan F. (2016). Minocycline protects against NLRP3 inflammasome-induced inflammation and P53-associated apoptosis in early brain injury after subarachnoid hemorrhage. *Mol. Neurobiol.* 53 2668–2678. 10.1007/s12035-015-9318-8 26143258

[B23] Li Q. Peng Y. Fan L. Xu H. He P. Cao S. (2018). Phosphodiesterase-4 inhibition confers a neuroprotective efficacy against early brain injury following experimental subarachnoid hemorrhage in rats by attenuating neuronal apoptosis through the SIRT1/Akt pathway. *Biomed. Pharmacother.* 99 947–955. 10.1016/j.biopha.2018.01.093 29710495

[B24] McBurney M. W. Yang X. Jardine K. Hixon M. Boekelheide K. Webb J. R. (2003). The mammalian SIR2alpha protein has a role in embryogenesis and gametogenesis. *Mol. Cell. Biol.* 23 38–54. 10.1128/MCB.23.1.38-54.2003 12482959 PMC140671

[B25] Menini S. Iacobini C. Ricci C. Oddi G. Pesce C. Pugliese F. (2007). Ablation of the gene encoding p66Shc protects mice against AGE-induced glomerulopathy by preventing oxidant-dependent tissue injury and further AGE accumulation. *Diabetologia* 50 1997–2007. 10.1007/s00125-007-0728-7 17611735

[B26] Miller D. M. Singh I. N. Wang J. A. Hall E. D. (2015). Nrf2-ARE activator carnosic acid decreases mitochondrial dysfunction, oxidative damage and neuronal cytoskeletal degradation following traumatic brain injury in mice. *Exp. Neurol.* 264 103–110. 10.1016/j.expneurol.2014.11.008 25432068 PMC4323924

[B27] Miyazawa M. Tsuji Y. (2014). Evidence for a novel antioxidant function and isoform-specific regulation of the human p66Shc gene. *Mol. Biol. Cell* 25 2116–2127. 10.1091/mbc.E13-11-0666 24807908 PMC4072584

[B28] Olmez I. Ozyurt H. (2012). Reactive oxygen species and ischemic cerebrovascular disease. *Neurochem. Int.* 60 208–212. 10.1016/j.neuint.2011.11.009 22122807

[B29] Ostrowski R. P. Colohan A. R. Zhang J. H. (2006). Molecular mechanisms of early brain injury after subarachnoid hemorrhage. *Neurol. Res.* 28 399–414. 10.1179/016164106X115008 16759443

[B30] Park M. Y. Mun S. T. (2013). Dietary carnosic acid suppresses hepatic steatosis formation via regulation of hepatic fatty acid metabolism in high-fat diet-fed mice. *Nutr. Res. Pract.* 7 294–301. 10.4162/nrp.2013.7.4.294 23964317 PMC3746164

[B31] Purushotham A. Schug T. T. Xu Q. Surapureddi S. Guo X. Li X. (2009). Hepatocyte-specific deletion of SIRT1 alters fatty acid metabolism and results in hepatic steatosis and inflammation. *Cell Metab.* 9 327–338. 10.1016/j.cmet.2009.02.006 19356714 PMC2668535

[B32] Redza-Dutordoir M. Averill-Bates D. A. (2016). Activation of apoptosis signalling pathways by reactive oxygen species. *Biochim. Biophys. Acta* 1863 2977–2992. 10.1016/j.bbamcr.2016.09.012 27646922

[B33] Sahu B. D. Putcha U. K. Kuncha M. Rachamalla S. S. Sistla R. (2014). Carnosic acid promotes myocardial antioxidant response and prevents isoproterenol-induced myocardial oxidative stress and apoptosis in mice. *Mol. Cell. Biochem.* 394 163–176. 10.1007/s11010-014-2092-5 24903830

[B34] Sahu B. D. Rentam K. K. Putcha U. K. Kuncha M. Vegi G. M. Sistla R. (2011). Carnosic acid attenuates renal injury in an experimental model of rat cisplatin-induced nephrotoxicity. *Food Chem. Toxicol.* 49 3090–3097. 10.1016/j.fct.2011.08.018 21930180

[B35] Sehba F. A. Friedrich V. (2015). Early events after aneurysmal subarachnoid hemorrhage. *Acta Neurochir. Suppl.* 120 23–28. 10.1007/978-3-319-04981-6_4 25366594

[B36] Sehba F. A. Hou J. Pluta R. M. Zhang J. H. (2012). The importance of early brain injury after subarachnoid hemorrhage. *Prog. Neurobiol.* 97 14–37. 10.1016/j.pneurobio.2012.02.003 22414893 PMC3327829

[B37] Shan W. Gao L. Zeng W. Hu Y. Wang G. Li M. (2015). Activation of the SIRT1/p66shc antiapoptosis pathway via carnosic acid-induced inhibition of miR-34a protects rats against nonalcoholic fatty liver disease. *Cell Death Dis.* 6 e1833. 10.1038/cddis.2015.196 26203862 PMC4650741

[B38] Sugawara T. Ayer R. Jadhav V. Zhang J. H. (2008). A new grading system evaluating bleeding scale in filament perforation subarachnoid hemorrhage rat model. *J. Neurosci. Methods* 167 327–334. 10.1016/j.jneumeth.2007.08.004 17870179 PMC2259391

[B39] Venditti P. Di Stefano L. Di Meo S. (2013). Mitochondrial metabolism of reactive oxygen species. *Mitochondrion* 13 71–82. 10.1016/j.mito.2013.01.008 23376030

[B40] Vikram A. Kim Y. R. Kumar S. Naqvi A. Hoffman T. A. Kumar A. (2014). Canonical Wnt signaling induces vascular endothelial dysfunction via p66Shc-regulated reactive oxygen species. *Arterioscler. Thromb. Vasc. Biol.* 34 2301–2309. 10.1161/ATVBAHA.114.304338 25147340 PMC6069972

[B41] Wang G. Z. Yao J. H. Jing H. R. Zhang F. Lin M. S. Shi L. (2012). Suppression of the p66shc adapter protein by protocatechuic acid prevents the development of lung injury induced by intestinal ischemia reperfusion in mice. *J. Trauma Acute Care Surg.* 73 1130–1137. 10.1097/TA.0b013e318265d069 23117377

[B42] Wang P. Xu T. Y. Guan Y. F. Tian W. W. Viollet B. Rui Y. C. (2011). Nicotinamide phosphoribosyltransferase protects against ischemic stroke through SIRT1-dependent adenosine monophosphate-activated kinase pathway. *Ann. Neurol.* 69 360–374. 10.1002/ana.22236 21246601

[B43] Wu P. Li Y. Zhu S. Wang C. Dai J. Zhang G. (2017). Mdivi-1 alleviates early brain injury after experimental subarachnoid hemorrhage in rats, possibly via inhibition of Drp1-activated mitochondrial fission and oxidative stress. *Neurochem. Res.* 42 1449–1458. 10.1007/s11064-017-2201-4 28210956

[B44] Xia P. Pan Y. Zhang F. Wang N. Wang E. Guo Q. (2018). Pioglitazone confers neuroprotection against ischemia-induced pyroptosis due to its inhibitory effects on HMGB-1/RAGE and Rac1/ROS pathway by activating PPAR. *Cell Physiol. Biochem.* 45 2351–2368. 10.1159/000488183 29554649

[B45] Xie Y. Zhang J. Ye S. He M. Ren R. Yuan D. (2012). SirT1 regulates radiosensitivity of hepatoma cells differently under normoxic and hypoxic conditions. *Cancer Sci.* 103 1238–1244. 10.1111/j.1349-7006.2012.02285.x 22448750 PMC7659182

[B46] Yan F. Cao S. Li J. Dixon B. Yu X. Chen J. (2016). Pharmacological inhibition of PERK attenuates early brain injury after subarachnoid hemorrhage in rats through the activation of Akt. *Mol. Neurobiol.* 54 1808–1817. 10.1007/s12035-016-9790-9 26887383

[B47] Yan H. Jihong Y. Feng Z. Xiaomei X. Xiaohan Z. Guangzhi W. (2014). Sirtuin 1-mediated inhibition of p66shc expression alleviates liver ischemia/reperfusion injury. *Crit. Care Med.* 42 e373–e381. 10.1097/CCM.0000000000000246 24557422

[B48] Zaccagnini G. Martelli F. Magenta A. Cencioni C. Fasanaro P. Nicoletti C. (2007). p66(ShcA) and oxidative stress modulate myogenic differentiation and skeletal muscle regeneration after hind limb ischemia. *J. Biol. Chem.* 282 31453–31459. 10.1074/jbc.M702511200 17726026

[B49] Zhang L. Wu J. Duan X. Tian X. Shen H. Sun Q. (2016). NADPH oxidase: a potential target for treatment of stroke. *Oxid. Med. Cell. Longev.* 2016:5026984. 10.1155/2016/5026984 26941888 PMC4752995

[B50] Zhang X. S. Wu Q. Wu L. Y. Ye Z. N. Jiang T. W. Li W. (2016). Sirtuin 1 activation protects against early brain injury after experimental subarachnoid hemorrhage in rats. *Cell Death Dis.* 7:e2416. 10.1038/cddis.2016.292 27735947 PMC5133967

[B51] Zhou S. Chen H. Z. Wan Y. Z. Zhang Q. J. Wei Y. S. Huang S. (2011). Repression of P66Shc expression by SIRT1 contributes to the prevention of hyperglycemia-induced endothelial dysfunction. *Circ. Res.* 109 639–648. 10.1161/CIRCRESAHA.111.243592 21778425

